# Acromion type III scapular spine nonunion open reduction internal fixation with distal clavicular and pelvic plates

**DOI:** 10.1016/j.xrrt.2026.100761

**Published:** 2026-04-30

**Authors:** Anna L. Gorsky, Jesse Seilern und Aspang, Kier M. Blevins, Krishna N. Chopra, Michael B. Gottschalk, Eric R. Wagner

**Affiliations:** Department of Orthopaedic Surgery, Emory University School of Medicine, Atlanta, GA, USA

**Keywords:** Fracture, Acromion, Scapular spine, Nonunion, Dual plating, Clavicle plate, Pelvic reconstruction plate, Open reduction internal fixation

Acromion fractures are a known complication of reverse shoulder arthroplasty (rTSA), with the incidence of these injuries reported from 1%-11%.^21^^,^^24^^,^^29^ These fractures contribute to significant morbidity in the post-operative setting, including pain, dysfunction, and an increased rate of revision arthroplasty.^24^^,^^27^ The Levy classification categorizes acromion fractures into 3 types based on location and involvement of deltoid origin.^16^ Levy types I and II are typically appropriate for a trial of nonoperative management, while type III fractures are less likely to succeed without surgical fixation.^16^ Conservative management is generally appropriate for stable, nondisplaced acromion fractures; however, surgical intervention is typically warranted for unstable or displaced fractures and those that fail to heal with nonoperative management. A substantial proportion of these injuries progress to nonunion,^10^^,^^18^^,^^20^^,^^21^ thereby necessitating surgical fixation to restore function and mitigate pain.

There are a variety of surgical techniques described for surgical fixation of acromion fractures. Tension band wiring, while historically used, has been associated with inconsistent outcomes.^27^^,^^28^ More recently, plate and screw fixation have demonstrated promising results in terms of pain relief and functional improvement^7^^,^^14^; however, a universally accepted standard of care has yet to be established. Despite these advancements, surgical management is not uniformly successful, with documented complications including hardware failure, nonunion, and increased rates of revision arthroplasty.^2^^,^^15^^,^^24^ Furthermore, post-operative patient-reported outcome measures are inconsistent.

We present a technique for open reduction internal fixation of an acromion type III^16^ scapular spine fracture nonunion following rTSA using superior clavicular and pelvic reconstruction plates in a dual plating construct. This technique provides increased stability, as the unique contour of the acromion is more effectively accommodated by the design of these modified implants.

## Clinical presentation

**Figure 1:**
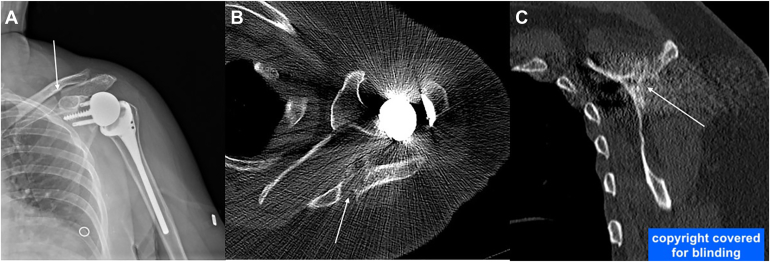
Pre-operative imaging. (**A**) Grashey view X-ray of the acromion fracture 4 weeks after injury, demonstrating the rTSA to be in adequate position. (**B**) Coronal view CT scan demonstrating the acromion fracture and rTSA 16 weeks after injury. (**C**) Sagittal view CT scan 16 weeks after injury. *rTSA*, reverse shoulder arthroplasty; *CT*, computed tomography.

A 67-year-old female presented to our clinic with acute shoulder pain and loss of motion after recurrent falls. Initial imaging demonstrated a well-positioned rTSA and nondisplaced Levy type III fracture of the acromion.^16^ The patient opted for nonoperative management and was placed in an abduction sling for 12 weeks. She continued to have pain, and a computed tomography scan at 16 weeks demonstrated a displaced nonunion with no evidence of potential bone healing (Fig. 1); therefore, the patient underwent operative fixation of the acromion.

## Technique

### Patient position and anesthesia

**Figure 2:**
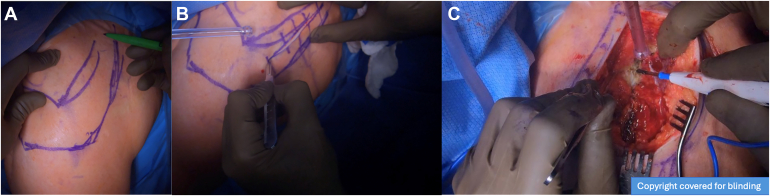
The approach to the posterior acromion. (**A**) The patient is set up in a beach chair position, and the anatomy of the acromion is marked as a guide. (**B**) The incision is made over the posterior acromion and scapular spine. (**C**) The interval is developed between the deltoid and trapezius, and the origins/insertions are elevated off the scapular spine.

This outpatient procedure uses general and regional anesthesia. The patient is placed in a beach chair position, and a transverse incision is centered over the posterior acromion and scapular spine (Fig. 2). A modified beach chair^3^ or true lateral decubitus^17^ position may also be appropriate; positioning is determined based on surgeon preference and location of the fracture.

### Surgical approach and fracture reduction

**Figure 3:**
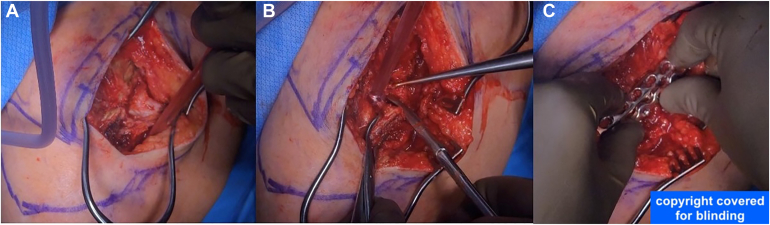
Visualization and débridement of the fracture nonunion site. (**A**) The fracture site is identified toward the base of the acromion, consistent with the imaging as a Levy type III. (**B**) The nonunion is débrided with a curette and rongeur for enhanced visualization and preparation of the bone. Lobster clamps are used to manipulate the fracture segments during débridement and subsequently for reduction. (**C**) Once reduction is provisionally achieved, the plates are tested to determine fit and contour over the fracture site.

The skin is incised in layered fashion, and hemostasis is achieved using electrocautery. Upon identification of the underlying fascia, the trapezius and deltoid musculature are visualized as they converge along the superior aspect of the scapular spine. The interval between these muscles is carefully developed (Fig. 2), and their insertions/origins are elevated from the scapular spine to allow for fracture exposure. The dissection is carried proximally and distally until the fracture site is identified (Fig. 3). The nonunion site is then thoroughly débrided using a curette and rongeur to remove fibrous tissue and prepare the bony surfaces (Fig. 3). Once adequate visualization is achieved, fracture reduction is performed with 2 lobster clamps to manipulate and align the fragments.

### Implant fixation

**Figure 4:**
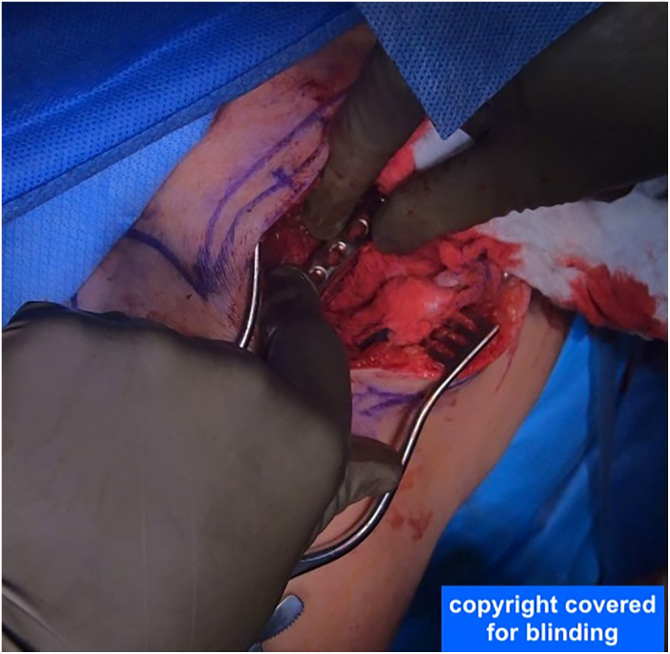
Contouring the superior clavicle plate. The plate is placed over the fracture site and fitted to the anatomy of the acromion. Bending pliers are used to contour the plate, which may take 2 to 3 attempts to achieve the exact curvature.

**Figure 5:**
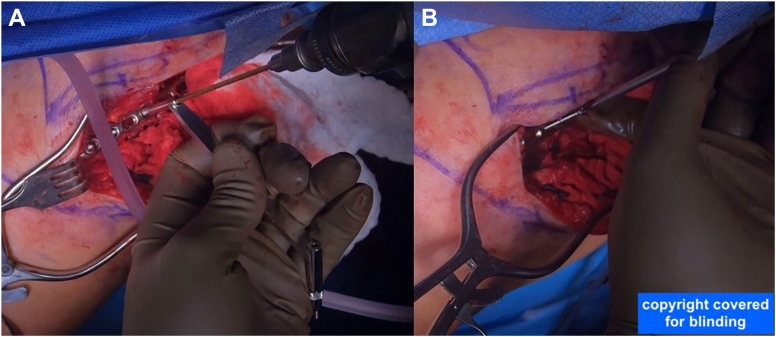
Clavicle fixation over the posterior acromion. (**A**) The superior clavicle plate is placed over the posterior aspect of the acromion and held in place with a lobster clamp while drilling. (**B**) The lateral fragment of the plate is filled with locking and nonlocking screws. Nonlocking screws are then placed proximally to compress the fracture site.

**Figure 6:**
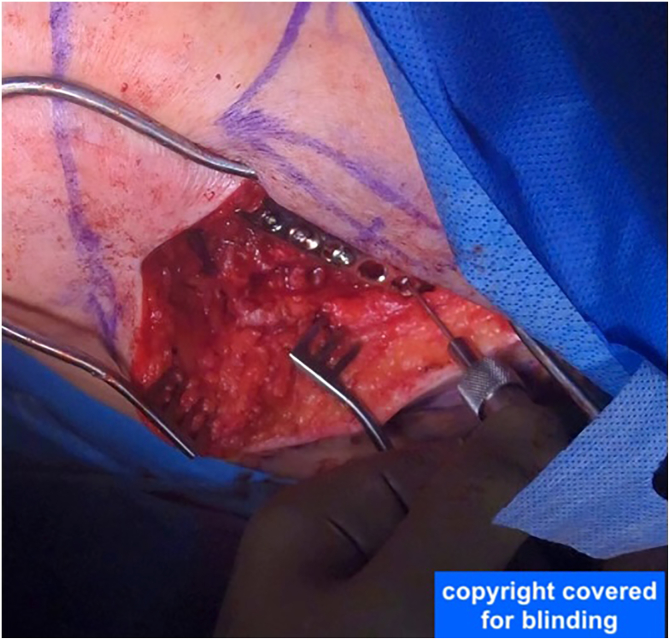
Final screws of the first plate. Proximal screws are placed to compress across the fracture site with the superior clavicle plate. Due to the high mechanical demands of the upper extremity, a single plate was deemed inadequate for a stable construct.

**Figure 7:**
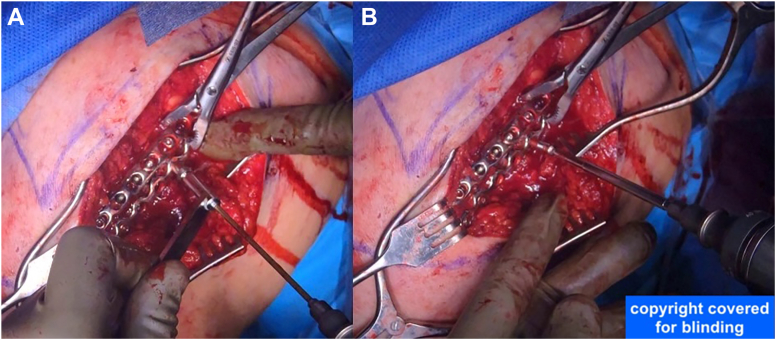
Pelvic reconstruction plate application. (**A**) After completing the superior clavicle plate, a pelvic reconstruction plate is applied posteriorly and inferiorly in a perpendicular manner. (**B**) In the same fashion as the superior clavicle plate, distal fixation is secured with locking and nonlocking screws then followed by proximal nonlocking screws.

**Figure 8:**
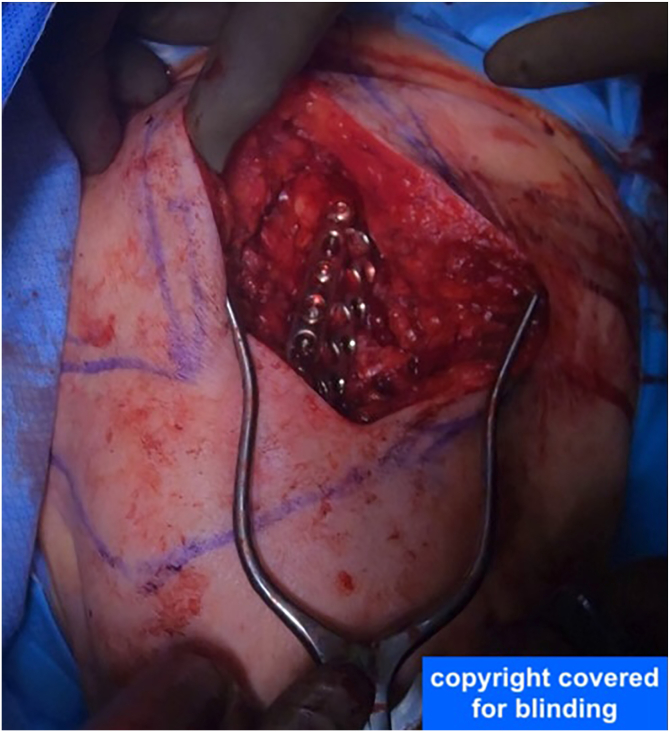
Final view of the dual plating construct. Once both plates are fixed, fluoroscopy is used to confirm the reduction and screw trajectory. The patient's shoulder is taken through an arc of motion prior to closure.

The 3.5-mm superior clavicle locking compression plate is selected first and contoured to match the posterior aspect of the scapular spine and acromion (Fig. 4). This implant type has been shown to yield the highest load to failure and most favorable strain profile when used in this position.^12^ Sequential drilling and screw placement of the lateral fragment is achieved first with a combination of locking and nonlocking screws (Fig. 5), followed by proximal nonlocking screws to achieve compression across the fracture site (Fig. 6). Due to high mechanical demands placed on the construct through the long lever of the upper extremity, a second plate is applied in a perpendicular (90-90) configuration. This combination has been shown to produce superior stiffness, higher load to failure, and less micromotion across the fracture site, when compared to single plate techniques.^11^ A 3.5-mm pelvic reconstruction plate is selected and contoured accordingly. This plate's versatile contouring ability allows for precise, patient-specific anatomical adaptation to complex and irregular surfaces, without jeopardizing the plate integrity. A combination of locking and nonlocking screws is used again for distal fixation, followed by proximal screw placement using the same sequence (Figs. 7 and 8). Biplanar fluoroscopy is used to confirm the reduction, screw trajectory, and plate placement. The C-Arm comes in from the opposite side of the bed and is positioned to allow for easy and efficient images, with the beam parallel to the scapular spine. The shoulder is taken through range of motion to confirm a stable arc of motion. The surgical site is then irrigated thoroughly, and vancomycin powder is applied prior to layered wound closure using interrupted sutures. A sterile dressing is applied, and the patient is placed in a gunslinger brace.

### Post-operative management

**Figure 9:**
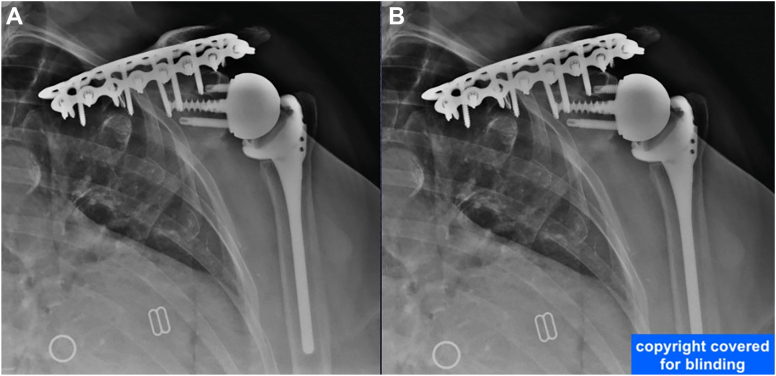
Post-operative images. Imaging at (**A**) 6 weeks and (**B**) 12 months post-operatively demonstrates stable implants.

Patients are immobilized in a gunslinger brace for 6 weeks. Scapular X-rays are obtained at the 6-week mark to confirm adequate alignment of the hardware (Fig. 9) and a computed tomography scan is performed at 3 months to assess bony healing. Passive and active-assisted motion begins at the 6-week mark when the patient transitions out of the sling and restrictions are lifted at 12 weeks while the patient continues to work on range of motion and strengthening.

## Discussion

We describe a technique for open reduction internal fixation of an acromial fracture nonunion after rTSA using perpendicular superior clavicle and pelvic reconstruction locking plates (Video 1). This perpendicular dual plate construct provides stable fixation that accommodates the complex acromial contour. In our case of a Levy type III nonunion that failed nonoperative care, the patient achieved radiographic union. At 18 months post-operatively, the patient had no pain, subjective shoulder value of 75%, and an American Shoulder and Elbow Surgeons score of 62, demonstrating significant improvement from her pre-operative status of visual analog scale pain 10 and extremely limited shoulder function.

Acromion and scapular spine fractures after rTSA are uncommon but clinically significant complications that impair function and increase the risk of revision surgery.^21^^,^^24^^,^^27^^,^^29^ Fixation is challenging because the deltoid imposes substantial deforming forces, and plate conformity to the acromion is often suboptimal.^1^^,^^11^ As the utilization of rTSA rises,^8^^,^^26^ the absolute number of these fractures is also expected to increase.^4^ Existing reports show mixed results with tension band constructs and generally better pain relief and function with plate fixation, yet no single technique has emerged as a standard of care.^5^^,^^7^^,^^14^^,^^27^^,^^28^ Proposed strategies include spanning the scapular spine with a single plate, incorporating hook features, and adding bone graft when indicated.^9^^,^^25^^,^^30^ Biomechanical and early clinical data suggest that dual plating across the acromion and scapular spine improves resistance to displacement under deltoid loading and is well suited to junctional and base patterns that experience high tensile forces.^11^^,^^12^^,^^22^^,^^23^ Dual plating techniques are not frequently used in patients with acromion fractures not related to an rTSA; however, there are biomechanical studies that suggest the dual plate construct demonstrates higher load-to-failure and stability.^11^^,^^19^ Implant selection is critical because a meaningful portion of acromion curvatures does not match conventional plates. Clavicle plates often better approximate the scapular spine, and pelvic reconstruction plates allow precise contouring, which together facilitate a rigid 90-90 construct with broad bony contact.^1^^,^^6^^,^^13^

### Indications and implant selection

Candidates for this technique include unstable or displaced acromion or scapular spine fractures and nonunions following rTSA that have not responded to nonoperative care, particularly junctional and base patterns that are subjected to high deltoid tension. Implant selection should prioritize plates that can be contoured to the posterior acromion and scapular spine, span the fracture zone, provide broad surface contact, and permit safe screw trajectories. When surface mismatch is a concern, clavicular plates tend to conform more reliably to the scapular spine, and pelvic reconstruction plates permit fine contouring. When used together, these implants can be contoured to create a rigid 90-90 construct that improves stability and load sharing.^1^^,^^6^^,^^13^

### Technical pearls and pitfalls

It is important to develop the trapezius to deltoid interval carefully to preserve soft tissues and facilitate reduction. Prepare the nonunion bed with thorough débridement to optimize biology, obtain provisional reduction, and achieve compression with nonlocking screws before augmenting with locking fixation. Precontour the clavicle plate along the posterior acromion and scapular spine and then apply the pelvic reconstruction plate perpendicularly to complete a true 90-90 frame that resists deforming forces and distributes load across broader contact areas.^1^ Confirm screw length and trajectory with biplanar fluoroscopy and reassess stability through a gentle arc of motion. Consider graft augmentation when bone quality is limited or a residual gap persists after débridement, as grafting can increase the likelihood of union in compromised scenarios.^9^^,^^25^

We found that a single plate did not provide the necessary stability around the fracture site, and thus, we opted for 2 plates in a 90-90 configuration, which we have now performed in 14 patients with considerable clinical success. For patients with type I or type II acromial fractures, we modify this technique to include a superior distal clavicle plate with a posterior pelvic reconstruction plate. The locked 90-90 double plate has been described in the literature with some success, but it typically uses 2 clavicular plates.^3^^,^^31^

This report has inherent limitations. It reflects a single patient’s outcome without comparative data and the risks of hardware prominence, plate mismatch, and nonunion persist, particularly in osteopenic bone. Broader series are needed to define union rates, complications, and functional recovery.

## Conclusion

A perpendicular dual plate construct using clavicle and pelvic reconstruction plates provides a stable, anatomically conforming option for acromial fracture nonunion after rTSA. Early experience is encouraging, but larger cohorts are required to validate indications, outcomes, and durability.

## Disclaimers:

Funding: No funding was disclosed by the authors.

Conflicts of interest: Eric R. Wagner reports that he has received consulting fees from Stryker, Smith and Nephew, Depuy-Synthes, and Acumed and institutional research support from Konica Minolta. He has also received hospitality fees from Arthrex, Wright, Stryker, Integra, and Acumed. None of these are relevant to this manuscript.

Michael B. Gottschalk reports that he has received institutional support from Skeletal Dynamics, Acumed, and Arthrex; research support from Stryker and Konica Minolta; and serves in editorial and organizational roles including as a board or committee member of the American Society for Surgery of the Hand, associate editor for the Journal of Hand Surgery, and Surgical Techniques in Orthopedics. He receives no royalties and none of these relationships are relevant to this manuscript.

Any additional authors, their immediate families, and any research foundations with which they are affiliated have not received any financial payments or other benefits from any commercial entity related to the subject of this article.
