# Supplementary material for: Acromion type III scapular spine nonunion open reduction internal fixation with distal clavicular and pelvic plates
Source: JSES Rev Rep Tech. 2026 Apr 30;6(3):100761. doi: 10.1016/j.xrrt.2026.100761 (PMC13265873; doi:10.1016/j.xrrt.2026.100761)
Supplement: Video Legend [file mmc2.docx]

**VIDEO LEGEND**

**Video 1:** Video demonstrating the technique for dual plating with superior clavicle and posterior pelvic reconstruction plates to attain open reduction and internal fixation of an acromion fracture nonunion in the setting of a reverse shoulder arthroplasty.
